# Endophthalmitis associated with *Staphylococcus cohnii* after vitrectomy and silicone oil insertion: A case report

**DOI:** 10.1097/MD.0000000000036574

**Published:** 2023-12-15

**Authors:** Yuqiu Zhang

**Affiliations:** a Department of Ophthalmology, The Second Hospital of Lanzhou University, Lanzhou, China.

**Keywords:** endophthalmitis, pars plana vitrectomy, silicone oil-filled eye, *Staphylococcus cohnii*

## Abstract

**Background:**

To report a case of endophthalmitis in a silicone oil (SO)-filled eye associated with *Staphylococcus cohnii*. After vitrectomy, the environment for bacterial growth in the eye is removed, and SO has antibacterial effect on a variety of microorganisms. Endophthalmitis is seen in about 0.040% cases after pars plana vitrectomy and is even more uncommon in cases where SO is used.

**Methods:**

The patient was diagnosed as endophthalmitis and admitted to our hospital for emergency. The main concern is if intraocular infection can be controlled and the visual prognosis. In this case, multiple intravitreal antibiotics injection and anterior chamber washout were performed. Not only that, phacoemulsification was performed.

**Results:**

Hypopyon became less after 3 operations were performed. The infection was under control finally.

**Conclusion:**

To the best of our knowledge, it is the first report of *S. cohnii* endophthalmitis in an SO-filled globe of an middle-aged patient. It is important to treat infective endophthalmitis with antibiotics promptly. Delayed therapy may affect the visual prognosis.

## 1. Introduction

Pars plana vitrectomy (PPV) combined with silicone oil (SO) tamponade is a standard technique in the treatment of complicated retinal detachment (RD).^[1]^ The incidence of endophthalmitis following PPV with SO tamponade is relatively low, partly because SO has been found to exhibit antimicrobial activity, inhibiting several species of pathogens.^[2]^ In this report, we present the case of a young man who underwent PPV with SO to treat RD. He developed endophthalmitis 2 weeks after the operation, but the infection was successfully treated through several intravitreal (IV) injections of ceftazidime and vancomycin.

## 2. Case report

**Figure 1.**
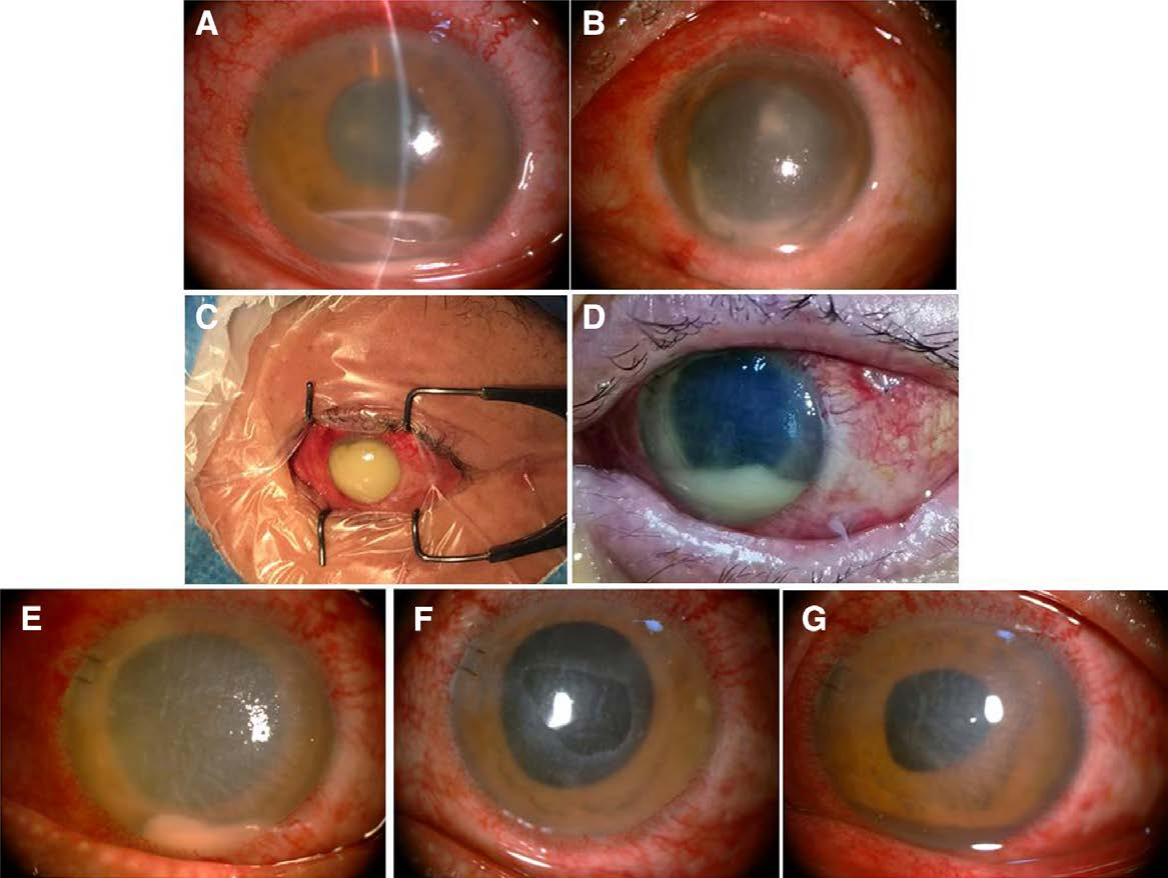
Photos of anterior segment before and after treatment in left eye. (A) November 7, a lot of fibrin exudation in anterior chamber and hypopyon; (B) November 8, the first day after the first intraocular injection, hypopyon; (C) November 11, 2 days after the second intraocular injection, hypopyon much more serious and prepare for the third injection; (D) November 12, the first day after the third intraocular injection, hypopyon again, chose to observe; (E) November 13, hypopyon decreased after 1 day; (F) November 18, hypopyon disappeared, corneal epithelium defected; (G) November 28, hypopyon disappeared, corneal epithelium healed.

A 49-year-old highly myopic male presented for a follow-up 17 days after PPV for a macular hole RD in his left eye (OS). He complained of decreased vision and puffiness lasting for 1 day. His previous history revealed no diabetes or trauma. The eye examination proceeded as follows: visual acuity was 20/40 right eye light perception (LP) OS and intraocular pressure was 13.4 mm Hg right eye 34.9 mm Hg OS. Under the slit lamp, it showed conjunctival congestion, corneal edema, exudate in the anterior chamber, and hypopyon OS (Fig. 1A). An ultrasound showed the retina was flat and attached. His vision was 20/500 OS 1 week following PPV surgery (October 28, 2019) and reduced to just LP OS 2 weeks afterward (November 7, 2019). The patient was diagnosed as endophthalmitis and admitted to our hospital for emergency. The same day, he underwent an operation for IV injections of vancomycin and ceftazidime and a subconjunctival injection of dexamethasone combined with atropine. A sample of aqueous humor was taken for bacterial and fungal culture. The following day (November 8, 2019), the pain in the OS worsened (Fig. 1B), and the patient was taken into the operating room again for an intraocular drug injection, similar to the first time. Fortunately, he felt better the next day, but hypopyon remained and even seemed slightly increased. The patient was treated with IV injection of vancomycin and ceftazidime after consultation with department of clinical pharmacology. But the patient had adverse effects such as rash when vancomycin was used. During this period, hypopyon became more and more as shown in Figure 1C (November 11, 2019). The patient was taken to the surgery room for the third time and treated with an anterior chamber washout, the addition of dexamethasone to the anterior chamber, and simultaneous IV injection of fluconazole. The anterior capsular was found ruptured after anterior chamber washout and phacoemulsification was performed. Again came that hypopyon the following day, but not much (Fig. 1D). The day after that there are encouraging signs that hypopyon became less as shown in anterior photo (Fig. 1E, F, G). A total of 3 operations were performed. The result from the bacterial and fungal cultures revealed the presence of *Staphylococcus cohnii*.

## 3. Ethical conduct statement

This study was conducted in compliance with the Declaration of Helsinki. The design of the study was approved by the appropriate Ethics Review Board of The Second Hospital of Lanzhou University, and the patient provided informed consent for publication of this report.

## 4. Discussion

Infectious endophthalmitis is a grave ophthalmic emergency characterized by sharp, rapidly progressing inflammation of the eye, primarily caused by fungi, bacteria, and parasites. If treatment is not administered in a timely manner, infection may spread to the entire eyeball and orbit, resulting in severe visual impairment. Should the opportunity for treatment be missed, antibiotics and surgery will prove ineffective, and ultimately the eye may not be preserved. Vitrectomy has been recognized as a safe and effective method to treat endophthalmitis.^[1,3]^ Vitrectomy removes the “medium” for bacterial growth and reproduction, as well as most bacteria and various toxins. And SO inhibits bacterial and fungal growth.^[4]^ On the one hand, SO is toxic to cell membrane, and on the other hand, it can deprive nutrients, resulting in microbial nutrient deficiency.^[3]^ Therefore, vitrectomy with SO filling can reduce the incidence of endophthalmitis.^[5,6]^ However, in recent years, due to improvements in surgical techniques, PPV cases have demonstrated a very low incidence rate of endophthalmitis, ranging from 0.03% to 0.04%, with the occurrence after SO filling being even rarer.^[7,8]^

In the case presented here, endophthalmitis occurred in the state of SO filling after vitrectomy, a condition with an exceptionally low incidence. The infection was confined to the eye, as there was no evidence of liver abscess on abdominal ultrasonography. The patient had no history of diabetes and was in good health. Endogenous endophthalmitis was excluded. Culture results identified the pathogen as a subspecies of *Staphylococcus cocci*, which is gram-positive and mainly includes the uremia and Koch subspecies. *Staphylococcus* is one of the most common pathogens in hospital infection^[9]^ and suppurative infections, which is suitable to settle in human skin, nose, throat, and so on, causing blood, respiratory tract, skin, and soft tissue infection.

The limitation of this study was that the cause or pathway of endophthalmitis in the case report was not very clear. It was clear that bacterial culture was positive, but we know that the incidence of endophthalmitis was very low after vitrectomy, especially in patients filled with SO. The patient experienced eye discomfort 14 days after surgery and found endophthalmitis. There was no timely follow-up 7 days after surgery, and it was unknown if there were any signs of infection. If detected earlier, perhaps the infection could be controlled more promptly and more vision could be preserved.

## 5. Conclusions

The patient was promptly treated for endophthalmitis with multiple intraocular injections and systemic antibiotic administration. Best of all, the condition was successfully controlled, and the prognosis for his vision was satisfactory. It has been reported that about 37.5% of patients with infectious endophthalmitis ended up with vision loss. However, the use of multiple IV and peribulbous injections of antibiotics and dexamethasone can reduce the likelihood of enucleation of the globe in patients with endogenous endophthalmitis.^[10]^ Unfortunately, the visual prognosis for infective endophthalmitis is generally poor. Therefore, it is crucial to treat infective endophthalmitis with antibiotics promptly. Any delay in therapy may adversely affect the visual prognosis.

## Author contributions

**Conceptualization:** Yuqiu Zhang.

**Data curation:** Yuqiu Zhang.

**Formal analysis:** Yuqiu Zhang.

**Project administration:** Yuqiu Zhang.

**Writing – original draft:** Yuqiu Zhang.

**Writing – review & editing:** Yuqiu Zhang.

## References

[1] Ashurov A Hundhammer M Sekundo W. Reasons and risk factors for recurrent retinal detachment after removal of silicon oil in various vitreoretinal diseases. Ophthalmologe. 2022;2:170–5. 10.1007/s00347-021-01420-6 34086072

[2] Sinisi F Della Santina M Loiudice P. The role of silicone oil in the surgical management of endophthalmitis: a systematic review. J Clin Med. 2022;11:5445. 36143089 10.3390/jcm11185445 PMC9505397

[3] Kaynak S Oner FH Koçak N. Surgical management of postoperative endophthalmitis: comparison of 2 techniques. J Cataract Refract Surg. 2003;5:966–9. 10.1016/s0886-3350(02)01892-8 12781284

[4] Ozdamar A Aras C Ozturk R. In vitro antimicrobial activity of silicone oil against endophthalmitis-causing agents. Retina. 1999;2:122–6. 10.1097/00006982-199902000-00006 10213237

[5] Abou Shousha M Eleiwa T Gibbons A. Risk of endophthalmitis in Boston type 1 keratoprosthesis combined with vitrectomy and silicone oil insertion. J Ophthalmol. 2019;2019:9648614. 31467698 10.1155/2019/9648614 PMC6701324

[6] Tabatabaei SA Soleimani M Vakili H. The rate of endophthalmitis after pars plana vitrectomy and its risk factors. Int Ophthalmol. 2019;39:1299–305. 29752592 10.1007/s10792-018-0944-9

[7] Eifrig CW Scott IU Flynn HW Jr. Endophthalmitis after pars plana vitrectomy: incidence, causative organisms, and visual acuity outcomes. Am J Ophthalmol. 2004;138:799–802. 15531315 10.1016/j.ajo.2004.06.035

[8] Bhende M Raman R Jain M. Incidence, microbiology, and outcomes of endophthalmitis after 111,876 pars plana vitrectomies at a single, tertiary eye care hospital. PLoS One. 2018;13:e0191173. 29338030 10.1371/journal.pone.0191173 PMC5770060

[9] Shengli L Yingyuan Z Jufang W. Investigation of staph nosocomial infections. J Chin Nosocomiol. 1998;8:65.

[10] Chen KJ Chen YP Chao AN. Prevention of evisceration or enucleation in endogenous bacterial panophthalmitis with no light perception and scleral abscess. PLoS One. 2017;12:0169603. 10.1371/journal.pone.0169603 PMC5215906 28056067

